# Scenario Analysis of Food Phosphorus Footprint in Kisumu, a Lakeside East African City in Lake Victoria (Kenya)

**DOI:** 10.3390/foods13142225

**Published:** 2024-07-16

**Authors:** Zheng Guo, Sophia Shuang Chen, Giri Raj Kattel, Wenyi Qiao, Linglong Lu, Rong Li, Anna Charles Mkumbo

**Affiliations:** 1 School of Geographical Sciences, Nanjing University of Information Science & Technology, Nanjing 210044, China; 003583@nuist.edu.cn (Z.G.); gkattel@unimelb.edu.au (G.R.K.); qiaowenyidl@163.com (W.Q.); 202183670017@nuist.edu.cn (L.L.); lirong020129@163.com (R.L.); 2 Key Laboratory of Watershed Geographic Sciences, Nanjing Institute of Geography and Limnology, Chinese Academy of Sciences, Nanjing 210008, China; 3 Department of Infrastructure Engineering, The University of Melbourne, Melbourne 3052, Australia; 4 Department of Hydraulic Engineering, Tsinghua University, Beijing 100190, China; 5 Tanzania Fisheries Research Institute, Dar es Salaam P.O. Box 750, Tanzania; annamkumbo49@gmail.com

**Keywords:** phosphorus footprint, food system, substance flow analysis, phosphorus management, east African city

## Abstract

Increased food production and consumption patterns have resulted in higher urban food phosphorus footprints, leading to a series of resource and environmental problems worldwide. We quantified the food phosphorus footprint of the African city of Kisumu using substance flow analysis. Our aim was to develop Kisumu’s sustainable phosphorus management framework so that the city would reduce phosphorus losses into the food system. Our results show that in the year 2023, the import and export of food phosphorus in the Kisumu food system was 2730.26 ± 2.7% t P yr^−1^ and 3297.05 ± 2.4% t P yr^−1^, respectively. There was −566.79 ± −18% t P yr^−1^ food phosphorus deficit in the Kisumu food system. Crop planting subsystem runoff/leaching/erosion loss, household consumption subsystem waste loss, and pit latrine subsystem blackwater loss are the major pathways of phosphorus losses into the environment and the main contributors to the food phosphorus footprint in the city. The 2030 scenario analysis shows that implementing a comprehensive scenario scheme throughout the entire lifecycle process from phosphorus input to waste disposal is the best choice for reducing phosphorus losses and suppressing the growth of food phosphorus footprint in the future. Our study shows that the food phosphorus footprint in the Kisumu food system was 0.67 kg P cap^−1^yr^−1^ in 2023, which is still at a low level but may enter a continuous upward trend with the improvement of socio-economic development of the city. In our framework, we have proposed a few essential measures that include urine separation, installation of septic tank, adjustment of dietary structure, flexible layout of sanitary disposal facilities, and separation of organic waste streams to reduce food phosphorus footprints in Kisumu. Given the similarity of cities along the shores of Lake Victoria, our calculation methods and management strategies can be applied to other cities in the region.

## 1. Introduction

Application of phosphorous fertilizers in agricultural fields around the world has greatly contributed to crop production and global food security [1]. Being a limited and rarely available non-renewable resource, the nutrient phosphorous has become a potentially important element in food production and consumption processes worldwide. Rapid urbanization during the 21st century has significantly increased food phosphorus consumption and emissions worldwide, consequently altering the often naturally intact phosphorus cycle in urban areas [2,3]. Scholars argue that the modified phosphorous cycle in urban areas is likely to result in two major overarching problems among residents. Firstly, this may lead to a reduction in the supply as a resource, and secondly, it may induce eutrophication due to excessive use in human food production and consumption [4].

A recent United Nations Environment Program (UNEP) resolution highlights improved management and cooperation in nutrients including phosphorus in urban areas worldwide [5], so that urban ecosystems and waterways are kept ecologically healthy and sustainable [6]. Today, how growing human activities interfere with the natural phosphorus cycle and its adverse impacts on urban environments and waterways, and how the degraded urban ecosystems could be restored and sustainably managed have become fundamentally significant to address urgently. Several studies in the past attempted to address food phosphorous footprint management in urban systems by using a range of methods. For instance, the substance flow analysis (SFA) approach, used initially to assess process, structure, and environmental emission pathways of food in the phosphorus cycle in Wuwei County (China), failed to deliver a perfect outcome due to variations in individual datasets [7], and quantitative approaches being used [8,9]. Hence, estimation of the food phosphorus footprint or the per capita phosphorus mass (kg P cap^−1^yr^−1^) emitted into the environment from the life cycle of food consumption by residents may vary widely depending on the methods being used for individual studies [10,11,12].

There are mainly two types of methods being used for calculating food phosphorus footprint. The phosphorus calculator (P-Calculator) method estimates food consumption phosphorus footprint and food production phosphorus footprint, where primarily, the consumption of each food is multiplied by its corresponding phosphorus content to obtain the food consumption phosphorus footprint [13]. The phosphorus footprint of food consumption is then multiplied by the virtual phosphorus factor (the phosphorus coefficient lost to the environment during food production and before food is consumed) to obtain the phosphorus footprint of food production [14]. Then, the food phosphorus footprint is eventually obtained [11]. For instance, combining food consumption data released by the Food and Agriculture Organization (FAO) and virtual phosphorus factors studied by Oita et al. [11] and Elrys et al. [13], Wirasenjaya et al. [15] estimated the contribution of different foods to food phosphorus footprint in Indonesia, where they found the footprint to be in an increasing trend from 2.13 kg P cap^−1^yr^−1^ in 2013 to 2.35 kg P cap^−1^yr^−1^ in 2030. However, the P-Calculator method alone cannot display the complete picture of the phosphorus cycle in the urban system, as the method does not address the key phosphorous reduction measures [16]. Unlike the P-calculator, the second SFA method evaluates the characteristics of urban phosphorus cycling with per capita phosphorus mass released into the environment at each stage of food production and consumption, followed by estimation of the food phosphorus footprint [17]. Given the improved performance, the SFA method has been applied in different countries, including Finland, Belgium, and USA. For instance, Grönman et al. [18] and Joensuu et al. [19] calculated food phosphorus footprint by assessing the phosphorus cycle in the production and consumption process of oats (0.006 kg P yr^−1^) and beef (0.049 kg P yr^−1^) in Finland, while Papangelou et al. [16] quantified the phosphorus cycle by assessing the phosphorus cycling process in food production and consumption and waste management in Brussels (7.7 kg P cap^−1^yr^−1^), Belgium (5.2 kg P cap^−1^yr^−1^), and the US (6.1 kg P cap^−1^yr^−1^). Although most of these studies systematically provided important measures to improve quantification of food phosphorous footprint and phosphorous recovery and utilization efficiency in richer countries, there has been a fundamental problem for the estimation of food phosphorous footprint in developing countries, such as Kenya (Africa), due to poor access to research data and limited research funding. In addition, the background values of nutrients and composition in the food, and a comparative global level of food phosphorus footprint in urban settings are still not fully established with clarity in Africa. Apart from a few studies, which have considered the impact of changes in urban population, consumption structure, and waste management and treatment technology on the food phosphorus footprint in Africa, most studies are based on phosphorous flow [20], and often rely on literature search data with limited certainty [21]. Hence, it is important to minimize uncertainty through reliable quantification of food phosphorous footprint in African cities.

Population growth and urbanization are the two major reasons causing African cities to face enormous resource and environmental challenges in the 21st century [22]. With the arrival of the second wave of urbanization by 2030, most of the world’s population growth in the coming decades is expected to occur in the coastal and Great Lakes lakeside areas of low- and middle-income countries in Africa [23,24]. Many residential areas in African countries are densely populated and rapidly expanding with spiraling poverty, inequality, informality, and spatial fragmentation of resources [25], indicating serious environmental consequences [26]. For example, the traditional food culture practices in African cities have been abandoned, with increased adoption of the multi-national food culture [27], resulting in the continuous increase in the food phosphorus demand followed by the food phosphorus footprint [28]. While the food phosphorous demand and footprint are on the rise, the corresponding waste management practices and capacity in African cities are becoming very weak, leading to a large amount of phosphorus being accumulated and lost in the system, consequently hampering the sustainability of national and regional ecological environments [20].

Kisumu, an east African city in Kenya, located in the edge of Lake Victoria, has recently experienced rapid urban growth and a range of environmental problems [23,29] with very high release of nutrients into the Winam Gulf, causing severe eutrophication and proliferation of an aquatic weed, the water hyacinth, in lake shores [30]. These nutrients are generated from a variety of anthropogenic sources, including agricultural production, residential consumption, and solid and liquid waste disposals [31,32]. Although agricultural runoff/leaching/erosion, animal manure loss, and urban waste loss are thought to be the main sources of phosphorus nutrients entering Lake Victoria, the specific point sources and the amounts of phosphorus nutrients produced by various activities have not yet been investigated for Kisumu [33,34].

A framework on the dynamics of nutrients including the food phosphorous footprint in the African city and integrating this with the city’s human development index (HDI) is becoming an increasingly useful indicator of the city’s development. For instance, fitting of nutrition footprint and HDI composed of life expectancy, education level, and people’s overall quality of life presents an environmental Kuznets curve, where the condition of improved social and economic level of the city, the nutrition footprint curve, shows an inverted U-shaped trend [35,36]. However, until recently, the development stage of regional nutrition footprint, as well as the food phosphorus footprint development level of Kisumu and its position in the global context based on HDI have been largely unexplored. Therefore, the research questions of this study are as follows. (1) How can we construct a Kisumu food system phosphorus flow analysis model based on the SFA method, and determine the key points of phosphorus loss or accumulation in the system? (2) What is the composition of residents’ food phosphorus footprint and its changes in differential future scenarios? (3) What are the differences between the stage of food phosphorus footprint level and other research results, and how can we reduce food phosphorus footprint?

## 2. Materials and Methods

### 2.1. Study Site

**Figure 1:**
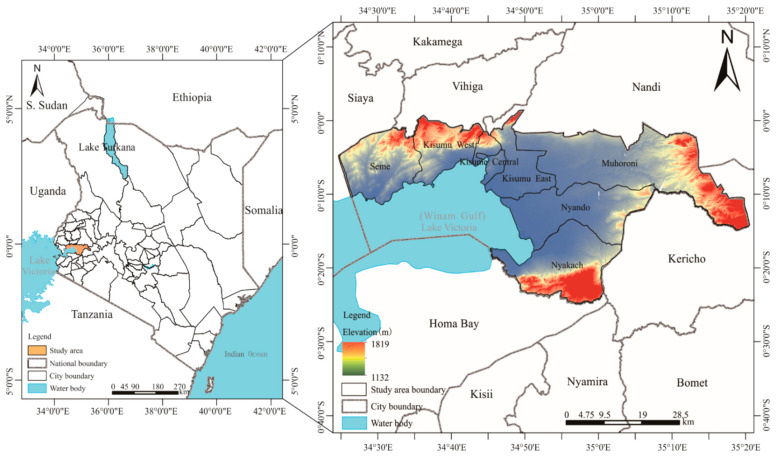
The map of Kisumu in Kenya, east Africa.

Kisumu, the third largest city, serves as the main economic and transportation center in western Kenya. Being in the equatorial (0°20′ S~0°50′ S and 33°20′ E~35°20′ E) region, and about 1132~1819 m above sea level, the city features unique geography and covers approximately 567 km^2^ water and 2085.4 km^2^ land area [37] (Figure 1). The major economic activities of Kisumu are farming, trade, and fishing, and the population of the city is 1,248,474 persons in the eight sub-regions [38]. Furthermore, Kisumu had the 5th highest average GDP/capita (USD 2029/capita) amongst Kenyan counties in the years 2015–2017 and is one of the most urbanized areas, where 50% of land is urbanized [29]. Today, Kisumu poses a severe challenge to the harmonious coexistence of people and natural ecosystems, due to the change in urban development towards high-speed and extensive modes of construction and transportation. For example, organic pollutants rich in phosphorus leaking into Lake Victoria cause eutrophication-related problems, including eutrophication and water hypoxia in the Winam Gulf of Lake Victoria [30].

### 2.2. Substance Flow Analysis

The substance flow analysis (SFA) method is an analytical tool that understands and characterizes the flow of specific substances (usually elements) within a specific system, and the whole system obeys the mass balance principle. An analysis system is defined by a set of elements including subsystems, flows, and stocks, as well as their interactions in terms of spatial (administrative district) and temporal (natural year) boundaries. The calculation methods can be classified into three kinds [7]: (a) calculating the flows or stocks by multiplying fixed activity level data and substance concentration parameters, for instance, F1; (b) quantifying flows or stocks based on other flows or stocks to obtain the relative quantities, for instance, F14; (c) accounting for the flows or stocks by balancing all flows or stocks of a subsystem, for instance, F11. Detailed information of the equations can be found in the Supplementary Material (Table S2).

**Figure 2:**
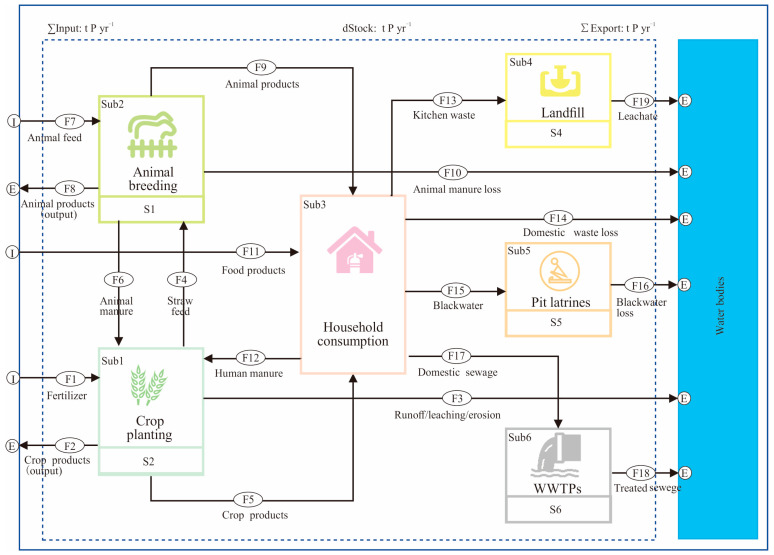
Food system phosphorus flow model of Kisumu, where I represents Input and E represents Export in the figure. The crop planting subsystem is the major consumer of phosphorus fertilizers and is also an important way for human and animal excrement to return to the phosphorus cycling network. The produced crop products are delivered to the household or outside Kisumu as food. In the meantime, the animal breeding subsystem obtains feed from the planting subsystem and outside Kisumu, and then produces animal products for household consumption; the waste disposal stage refers to the stage where phosphorus containing waste is treated through subsystems such as pit latrines, WWTPs, and landfills. Then, the phosphorus substance lifecycle in the Kisumu food system ends.

In this case, Kisumu, we quantified the phosphorus flow in the food system within the political boundaries of Kisumu in 2023. Based on our field assessment of the Kisumu food system, we finally identified six subsystems, nineteen flows, and five stocks (Figure 2). It is worth noting that some flows/subsystems were either added or dropped in this study, to suit the situation of the Kisumu food system. For example, although aquaculture and industrial sectors have some influences on phosphorus flow [20], they are not considered in the model in this study because the data are not available in an African city that could be compared. Generally speaking, to adapt to the characteristics of research cases, adding or reducing analysis system elements is currently a common practice [28]. The model calculation data and equations are detailed in the Supplementary Material (Tables S1 and S2).

### 2.3. Data Uncertainty and Error

In most existing SFA literature, the lack of sufficient, reliable, and region-specific high-quality data is considered a major obstacle to research in this field, affecting the accuracy and reliability of SFA results, and thus affecting the authority of research results in supporting relevant policies. Therefore, in the process of data collection in this study, special attention should be paid to issues related to data availability and quality, and the best data should be selected using a data collection method that is consistent with the actual situation and standardization of this study case. We adopted the uncertainty concept proposed by Hedbrant and Sörme [39] and referred to Klinglmair et al. [40]; Laner et al. [21] categorized data sources into 5 levels with different interval factor (Table 1). In this study, data in Level 1 were from the KNBS [38], e.g., city population, and are multiplied or divided by 1 (×÷1.02). Data in Level 2 were from internationally renowned institutions/organizations/journals, e.g., an average live weight of animals, and are multiplied or divided by 1.03 and so on, up to data in level 5, which were from expert estimates/educated people’s guesses.

For example, Kisumu has 1,248,474 persons, as reported by KNBS [38], and its uncertainty is ×÷1.02. Similarly, 60% of animal manure was estimated to return to cultivated soil by expert or educated guesses, and its uncertainty is ×÷1.12. In situations where datasets are being added or multiplied, the uncertainty was calculated using equations outlined by Antikainen et al. [41]. Data uncertainty increases when multiplying datasets and decreases when adding datasets.

Multiplication:

(1) $$Uncertainty\;factor=1+\sqrt{(f_{a}-1{)}^{2}+f_{b}-1{)}^{2}}$$

where $f_{a}$ and $f_{b}$ represent the assigned uncertainty interval for phosphorus flow 1 and flow 2, respectively.

For example, the cereal yield was 855,083,000 ×÷ 1.02 kg in Kisumu in 2023, and there are 2.22 ×÷ 1.03 g P kg^−1^product^−1^; the phosphorus in cereal was 1,898,284.26 kg.

Multiplication:

(2) $$Uncertainty\;factor=1+\sqrt{(1.02-1{)}^{2}+(1.03-1{)}^{2}}=1.04$$

Then, the final value of phosphorus will be 1,898,284.26 ×÷ 1.04

Addition:

(3) $$Uncertainty\;factor=1+\sqrt{\frac{{\left[m_{a}\times (f_{a}-1)\right]}^{2}+{\left[m_{b}\times (f_{b}-1)\right]}^{2}}{m_{a}+m_{b}}}$$

where $m_{a}$ and $m_{b}$ represent mass phosphorus 1 and 2, respectively.

For example, the phosphorus in cattle and goat manure is 2559 ×÷ 1.13 kg and 397 ×÷ 1.14 kg, respectively; then, we can calculate the uncertainty factor as follows:

(4) $$Uncertainty\;factor=1+\frac{\sqrt{{\left[2559\times (1.13-1)\right]}^{2}+{\left[397\times (1.14-1)\right]}^{2}}}{2559+397}=1.11$$

Then, the final value of phosphorus will be 2956 ×÷ 1.11

Furthermore, STAN 2.5 software was used in this study to modify the asymmetrical interval into a symmetric interval [21]; uncertain results can be seen in the Supplementary Material (Tables S1 and S2).

### 2.4. Definition of Food Phosphorus Footprint

Considering that one of the purposes of this study is to explore the impact of phosphorus emissions from the Kisumu food system on water bodies, we intend to define the food phosphorus footprint (FPF) in the Kisumu food system as the phosphorus loss into water bodies by each subsystem, that is, the phosphorus lost from the subsystems of crop planting (Sub1), animal breeding (Sub2), household consumption (Sub3), landfill (Sub4), pit latrines (Sub5), and WWTPs (Sub6) to the water bodies (Figure 2). The calculation principle is as follows:

(5) $$FPF=W_{Sub1}+W_{Sub2}+W_{Sub3}+W_{Sub4}+W_{Sub5}+W_{Sub6}$$

where FPF represents the food phosphorus footprint of the Kisumu food system; $W_{S\mathrm{ub}1}\sim W_{Sub6}$ represent the mass of phosphorus loss by each subsystem into the water bodies.

### 2.5. Scenarios and Indicators

Considering the data availability of Kisumu in the past year, 2023 is considered a reference scenario (defined as Scenario 0), and 2030 is chosen as the scenario analysis year due to its potential suitability for recent Kisumu planning and population forecasting. The scenarios’ contents are described below and summarized in Table 2.

Scenario 1: BAU (business as usual) scenario, only considering changes in population size; the population of Kisumu is expected to grow to 1,418,972 people by 2030 [42], with other variables changing with population changes.

Scenario 2: Vegetarian scenario, based on the BAU scenario, assuming that the dietary phosphorus intake of Kisumu residents follows the recommended phosphorus intake, namely close to the level of vegetarians [43]: from the current 2.1 g P cap^−1^day^−1^ to 1.5 g P cap^−1^day^−1^ in 2030. This assumption is because food consumption is the main source of phosphorus in the Kisumu food system.

Scenario 3: Kitchen waste scenario, based on the BAU scenario. We assumed that the ratio of Kisumu food waste collected would increase from the current 20% to 65% in 2030 for organic fertilizer production [44,45]. This scenario was inspired by Kalmykova et al. [46].

Scenario 4: Urine separation scenario, based on the BAU scenario. We assume that urine diversion measures are implemented in the pit latrine subsystem and reused in farmland. This situation was motivated by existing studies, which have shown that urine has enormous potential for human manure recycling in densely urbanized areas [47].

Scenario 5: Wastewater treatment scenario, based on the BAU scenario. We assume that the standard for phosphorus content in treated wastewater is 1 mg P L^−1^, This situation was motivated by the Kisumu Environmental Health and Sanitation Bill proposed by CGK [48].

Scenario 6: Waste incineration ash scenario, based on the BAU scenario. We assume that all phosphorus contained in food waste sent to landfills is recovered after incineration, to examine the max effects. This scenario was inspired by Wu et al. [49].

Scenario 7: Combined scenario. Vegetarian scenario, kitchen waste scenario, urine separation scenario, wastewater treatment scenario, and waste incineration scenario were combined.

## 3. Results

### 3.1. Food Phosphorus Flows in Kisumu Food System

**Figure 3:**
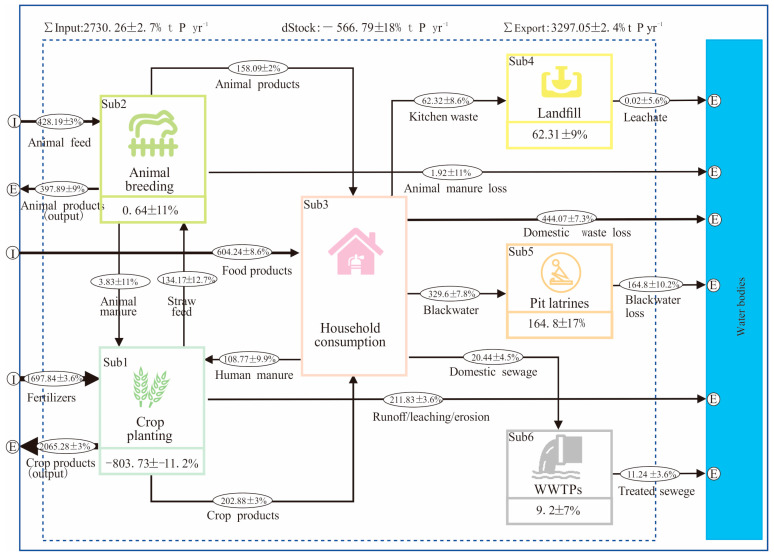
The food phosphorus flows in Kisumu food system (t P yr^−1^), where I represents Input and E represents Export in the figure. The main sources of phosphorus input into the Kisumu food system are fertilizer and food imports, while the main sources of phosphorus exports are animal and crop products. Crop planting subsystem runoff/leaching/erosion loss, household consumption subsystem waste loss, and pit latrine subsystem blackwater loss are the major pathways of phosphorus losses into the environment and the main contributors to the food phosphorus footprint. Subsystems of pit latrines and landfill were the two key nodes for phosphorus accumulation in the city.

In the year 2023, the food system of Kisumu imported approximately 2730.26 ± 2.7% t P/year, of which 1697.84 ± 3.6% t P yr^−1^ was fertilizers, 428.19 ± 3% t P yr^−1^ was animal feed, and 604.24 ± 8.6% t P yr^−1^ was other imported food products (Figure 3). In the same year, Kisumu exported approximately 3297.05 ± 2.4% t P yr^−1^ food, mainly crop and animal products, of which 2065.28 ± 3% t P yr^−1^ was crop product, and 397.89 ± 9% t P yr^−1^ was animal product export. Furthermore, considerable phosphorus was lost outside the food system, especially in crop soil runoff/leaching/erosion, domestic waste loss, blackwater discharge, and treated sewage, which were about 211.83 ± 3.6% t P yr^−1^, 164.8 ± 10.2% t P yr^−1^, 444.07 ± 7.3% t P yr^−1^, and 11.24 ± 3.6% t P yr^−1^ to the water bodies, respectively. Furthermore, the crop planting subsystem and animal breeding subsystem transported 134.17 ± 12.7% t P yr^−1^ and 3.83 ± 11% t P yr^−1^ to each other, achieving phosphorus recycling and reuse between these subsystem mutually. Moreover, these two subsystems made substantial inputs, 202.88 ± 3% t P/year and 158.09 ± 2% t P/year, to the household consumption subsystem, respectively. After food consumption from residents, approximately 329.6 ± 7.8% t P yr^−1^, 20.44 ± 4.5% t P yr^−1^, and 62.32 ± 8.6% t P yr^−1^ enters the waste disposal process, and approximately 108.77 ± 9.9% t P yr^−1^ enters the crop planting subsystem to meet the nutrient needs of plant growth in Kisumu. It is worth noting that an extra 803.73 ± 11.2% t P yr^−1^ was taken from the soil reserves every year, translating into 7.55 kg P ha^−1^yr^−1^ mined from the agricultural land, which is consistent with the conclusion drawn by Mnthambala et al. [28]. The stock of the pit latrine subsystem and landfill subsystem was highly considerable, as the phosphorous flows of 164.8 ± 17% t P yr^−1^ and 62.31 ± 9% t P yr^−1^ were well reflected, becoming important phosphorus sources for the recycling of phosphorus. In contrast, the stock of the animal breeding subsystem and WWTP subsystem was only 0.64 ± 11% t P yr^−1^ and 9.2 ± 7% t P yr^−1^, with relatively weak phosphorous flow in the system. The final food system’s phosphorus inflow was insufficient, resulting in the net stock of the food system being negative, at −566.79 ± 18% t P yr^−1^.

### 3.2. Food Phosphorus Footprint in Kisumu Food System

**Figure 4:**
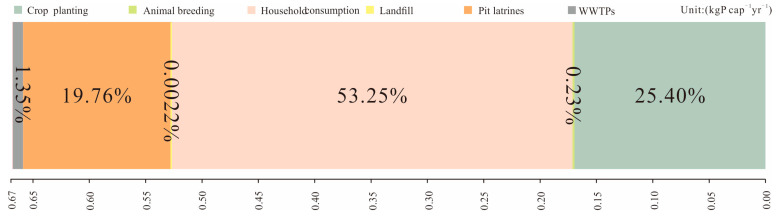
Food phosphorus footprint and its composition in Kisumu food system; the horizontal axis represents the food phosphorus footprint values generated by each subsystem, and the percentage number represents the proportion of food phosphorus footprint generated by each subsystem to the food phosphorus footprint of the food system.

Figure 4 shows that the food phosphorus footprint in the food system of Kisumu was 0.67 kg P cap^−1^yr^−1^ in 2023. More specifically, the contribution of each subsystem to the food phosphorus footprint varies significantly. The household consumption subsystem contributed 53.25% with 0.36 kg P cap^−1^yr^−1^ to the food phosphorus footprint. The reason is that the development scale of Kisumu’s health infrastructure lags far behind the urban population growth rate [48], and a large volume of domestic wastes, including kitchen wastes, and blackwater were lost to the water bodies during household use and consumption activity. The crop planting subsystem is a secondary component of the food phosphorus footprint, producing a food phosphorus footprint of 0.17 P cap^−1^yr^−1^, accounting for approximately 25.40%. This is primarily because the phosphorus application rate in Kisumu farmland is as high as 15.95 kg P yr^−1^ [50]. Secondly, there are topographic differences in the city, such as high terrain in the south and low terrain in the north, when the topographic conditions, combined with high annual precipitation (~max. 550 mm) [48], exacerbate the phosphorus loss from farmland to water bodies. Approximately 1.99 kg P ha^−1^yr^−1^ phosphorous is reported to be lost from farmland to the local waterways [51]. The food phosphorus footprint generated by the pit latrine subsystem is 0.13 kg P cap^−1^yr^−1^, accounting for approximately 19.76% in Kisumu. Many pit latrines in Kisumu lack seal, and the incidence of rainy season overflow is usually common. Further, human defecation and dumping in rivers are also a common practice, causing the high food phosphorus footprint in the pit latrine subsystem [20]. Unlike other subsystems, the food phosphorus footprint generated by the WWTP subsystem is relatively small, where only 1.35% or 0.01 kg P cap^−1^yr^−1^ food phosphorous footprint is generated. The amount of kitchen waste entering landfills is limited, and the amount of phosphorus lost through leaching is also small, so the proportion of phosphorus footprint for the WWTP subsystem generated was not significant.

### 3.3. Food Phosphorus Footprint in Different Scenarios in Kisumu Food System

**Figure 5:**
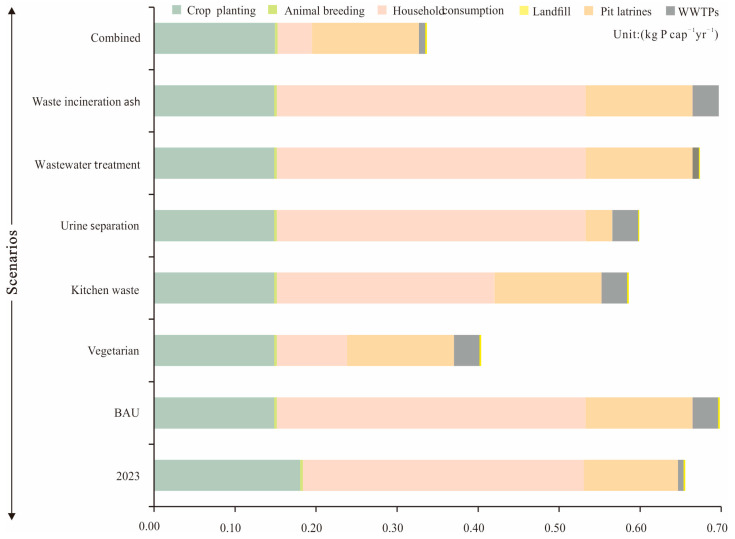
Yearly food phosphorus footprint with different scenarios for Kisumu, Kenya. The reference year is 2023 (Scenario 0) and the projected year is 2030 (Scenarios 1–6). For a description of the scenarios, see Section 2.5.

With 2023 as the reference scenario (0.67 kg P cap^−1^yr^−1^), the food phosphorus footprint in the BAU scenario will increase to 0.70 kg P cap^−1^yr^−1^ in Kisumu by 2030. More specifically (Figure 5), in the vegetarian scenario, the food phosphorus footprint decreased by 42.86% to 0.40 kg P cap^−1^yr^−1^ when compared to the BAU scenario, and even significantly lower than 0.67 kg P cap^−1^yr^−1^ in the 2023 scenario. In the kitchen waste scenario, there seem to be some benefits to the city’s environment due to the increase in the collection rates of kitchen waste from the municipality. The amount of phosphorus lost by the household consumption subsystem to Kisumu’s waterways has been found to be significantly reduced. The phosphorus footprint reduced from 0.70 kg P cap^−1^yr^−1^ in the 2023 reference scenario to 0.58 kg P cap^−1^yr^−1^ in the 2030 BAU scenario. Urine separation measures also have a significant impact on the reduction in the phosphorus footprint. The food phosphorus footprint decreased by 14.29% or 0.60 kg P cap^−1^yr^−1^ when comparing the 2023 reference scenario and the BAU scenario. In the wastewater treatment scenario, the food phosphorus footprint decreased to 0.68 kg P cap^−1^yr^−1^, indicating that the improvement of sewage treatment level has a relatively significant effect on the reduction in the food phosphorus footprint in Kisumu. Furthermore, the food phosphorus footprint in Kisumu is reduced from 0.70 kg P cap^−1^yr^−1^ in the BAU scenario to 0.69 kg P cap^−1^yr^−1^ in the waste incineration ash scenario; this is mainly because the limited phosphorus loss may have occurred from the landfill leachate [52]. It is worth noting that in the combined scenario, the food phosphorus footprint of the food system decreased to 0.34 kg P cap^−1^yr^−1^, and is only 58.62% and 50.75% in the combined scenario (2030) and reference scenario (2023), respectively.

## 4. Discussion

### 4.1. Global Comparison of Food Phosphorus Footprint

Food phosphorus footprint offers comparative analysis of research results among different geographic regions and scales. In our study, as far as possible, we chose the regions with different socio-economic development levels currently available as comparison objects to reflect the food phosphorus footprint level of Kisumu in the world. The Kisumu food phosphorus footprint is lower (0.67 kg P cap^−1^yr^−1^) than the food phosphorous footprint of Brussels, Luxembourg, the USA, Belgium, Japan, China, and India (Table 3), suggesting that cities in developing countries including Africa are still far behind in the consumption of food phosphorous. The lower food phosphorus footprint in Kisumu is due the city being lower in animal-based dietary consumption [53], as well as the limited use of phosphate fertilizer in agriculture [54]. The World Health Organization reported that Kisumu’s protein consumption is lower than the recommended intake of 75 g cap^−1^day^−1^ [55]. Study suggests that low food supply and low animal-based dietary consumption reduces phosphorus loss from the food consumed, and also during the production stages of foods with high protein content [13]. Being a small-scale food system, Kisumu has lower phosphorus losses during food production stages [14]. The food phosphorus footprints of some developing countries in Asia and Africa such as Indonesia (0.40 kg P cap^−1^yr^−1^) and Rwanda (0.31 kg P cap^−1^yr^−1^) are comparable to Kisumu, suggesting that people of cities in less developed economies have low dietary proteins. A very low food phosphorus footprint (1.60 kg P cap^−1^yr^−1^) of India compared to Brussels, Luxembourg, the USA, Belgium, Japan, and China strongly suggests that India, as a typical vegetarian country, consumes low animal protein [11]. Brussels has the highest food phosphorus footprint, followed by Luxembourg, the USA, Belgium, Japan, and China. These countries are not only richer economies, but they also consume more animal proteins with higher food phosphorous footprint [16].

**Figure 6:**
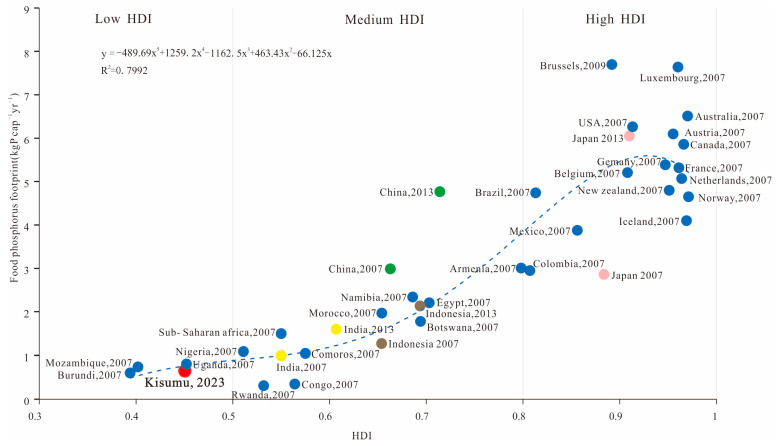
Trends in the relationship between food phosphorus footprint and HDI in different regions worldwide, with yellow, green, brown, and pink dots representing the food phosphorous footprint level of India, China, Indonesia, and Japan at different times, respectively. The red dots represent the food phosphorous footprint level of Kisumu, while the blue dots represent the food phosphorous footprint level of other counties in 2007 or 2009.

The global trend of food phosphorus footprint changes along with urbanization and socio-economic development (Section 4.1). When the food phosphorus footprint is fitted with the corresponding human development index (HDI) of countries, this shows two distinct stages: primarily the HDI shift, and the inverted U-shaped trend (Figure 6). When the HDI was between 0 and 0.9, the food phosphorus footprint of most regions worldwide increased along with the increase in the HDI, indicating a clear positive correlation between food phosphorus footprint and the HDI. However, when the HDI is in the range of 0–0.6, the food phosphorus footprint becomes relatively slow, mainly concentrating in the African continent, such as Burundi, Rwanda, Mozambique, as well as Kisumu. The HDI (0.6–0.9) reflects accelerated food phosphorus footprint due to improved living standard of the people [35]. Especially the changes in diets, both types and consumption pattern, such as shifting from vegetarian to animal-based diets, was eminent [11]. Developing countries such as Egypt, Indonesia, and China have all had a rapidly increasing trend of food phosphorous footprint. For instance, a global 3 kg P cap^−1^yr^−1^ food phosphorous footprint at 0.66 HDI has been found to increase to 6.05 kg P cap^−1^yr^−1^ at 0.71 HDI, indicating that the food phosphorus footprint of low-level cities, including Kisumu, may increase along with the socio-economic development in future. However, in the second stage, when the HDI exceeds 0.9, the food phosphorus footprint shows a downward trend, suggesting that restrictions in the excessive use of phosphorus and development of innovative technologies, such as chemical precision in agriculture, and biological phosphorus removal method would be paramount [57]. In addition, the European Union (EU), together with some representative countries, including Australia, Canada, France, the Netherlands, Norway, and Iceland, have proposed legislative amendments to phosphorus use, calling for a low-phosphorus diet and strengthening the recycling of organic wastes, to achieve a low-phosphorus future through green, renewable, or net-zero emission technologies worldwide [58].

### 4.2. Measures of Phosphorus Footprint

Although the current food phosphorus footprint of the Kisumu food system in our study was at a low level, it would still impact negatively on local waterways. With the city’s socio-economic development, the food phosphorus footprint in Kisumu may further accelerate. Hence, appropriate measures, such as the reuse and recycling of nutrients or a shift to a shorter and more local food system, should be adopted to reduce the food phosphorus footprint in Kisumu [16]. Closure in phosphorus import and recycling within food phosphorous subsystems is crucial for Kisumu. For instance, the subsystems, crop planting, animal breeding, and household consumption, when interacting with each other, only achieve partial phosphorus waste cycling. The phosphorus utilized in the crop planting subsystem is far from sufficient to compensate for the soil nutrient deficit caused by crop nutrient absorption (Figure 3). Hence, the waste generated from the animal breeding subsystem should be utilized for urban agriculture by constructing a closed loop of planting and breeding waste [59]. It is worth noting that about 108.77 ± 9% t P yr^−1^ phosphorus in human manure in Kusum is derived from the household consumption subsystem. The human manure is then utilized by the agricultural planting subsystem in the city. However, there is still as high as 329.6 ± 7.8% t P yr^−1^ entering pit latrines, where 50% of this lost to waterways in Kisumu (Figure 3), causing eutrophication and management challenges. Our scenario analysis results suggest that separating urine phosphorous from the pit latrine subsystem would be an efficient and cost-effective measure in Kisumu. The WHO pointed out that human urine is basically sterile, and if urine and manure are separated in the pit latrines [60], then the urine can be safely stored and used for agricultural production. Furthermore, measures such as the anaerobic biological treatment of human manure and urine have also been suggested to be useful for the region [3]. In the tropics, the bacterial activity is relatively high in waste decomposition processes, so anaerobic treatment could be effective for the mixture of urine and manure [61]. Installing a septic tank (ST) can effectively solve the household hygiene treatment system [62], and can be applied either separately to individual residential areas or centrally to residential communities. Shutting down crude pit latrines by replacing the ST system will greatly reduce the risk of phosphorus loss, thereby slowing down the growth of food phosphorus footprint in the city. Furthermore, a 42.86% reduction in the food phosphorus footprint in the vegetable scenario (Figure 5) strongly suggests that the adjustment of the dietary structure in the city, especially a decrease in animal-based product consumption or maintenance of nutrient intake as per the nutritional requirements of city people, is needed [56].

As mentioned earlier, the waste loss from the household consumption subsystem is the largest contributor to the phosphorus footprint of the Kisumu food system, which is largely caused by the scarcity of solid and liquid disposal facilities within the city. Buathong et al. [63] argue that in the construction process of urban waste disposal facilities in low-level developing countries, multiple flexible measures should be adopted. If the population density is high in the central area of Kisumu, constructing a centralized sewage treatment plant is the best solution for the inner urban area, while for the urban central edge area, the combination of centralized sewage treatment plants and decentralized sewage treatment plants may be the best choice. It has been argued that suburban areas cannot be integrated into the centralized sewage systems and treatment plants due to the distance and high construction costs; hence, constructing small-scale decentralized on-site sanitation systems is considered a feasible option for cities like Kisumu [64]. Our on-site investigation also found that the current waste entering the Kisumu landfills is mostly a mixture of multiple waste streams, which makes it difficult to recover phosphorus. Therefore, important resource streams related to phosphorus need to be separated and recycled separately to reduce the food phosphorus footprint [65]. The scenario analysis results in our study suggesting that developing a detailed framework in the implementation of a comprehensive scenario plan throughout the entire life cycle process from phosphorus input to waste disposal is the best choice to reduce phosphorus loss and avoid an increase in the food phosphorus footprint in Kisumu in the future. Furthermore, given the similarities in social, economic, cultural, and geographical characteristics of cities along Lake Victoria, we believe that these measures can also be used for phosphorus resource management and environmental protection in other rapidly urbanizing areas around the Lake Victoria basin.

### 4.3. Limitations and Implications of Model Choices

Sufficient baseline data are of great significance for accurately analyzing the phosphorus flow and developing effective and credible phosphorus management policies. However, there is a certain degree of uncertainty in our results due to the use of statistical data and parameter data from different sources. The data on phosphorus flow calculated by inputting relevant material flow quality and corresponding modeling were from different sources, and can have inherent uncertainties. To reduce uncertainty, we conducted statistical analysis in the reliability level distribution of the existing data (in Supplementary Material Table S1), as suggested by Hedbrant and Sörme [39]. We identified any uncertainty that remained in the phosphorus flow model of the Kisumu food system quantitatively (Figure 3 and Supplementary Material Table S2). Our study also identified priority fields for data collection. While constructing the phosphorus flow analysis model for the Kisumu food system, the complete subsystems for phosphorus flow were limited by data availability. For example, before crops or animal products enter the household consumption subsystem, there may be phosphorous leakages at the food processing plant subsystem, becoming an important source of phosphorus pollution in water bodies. Unlike other studies [16], no information related to food processing logistics in Kisumu was available, so we were not able to incorporate the food processing plant subsystem in our model. There were also shortcomings in the model, such as simplifying assumptions about the transfer process of phosphorus in the environment, which may have biased the results. For example, during the field investigation, we found that, apart from being collected into landfills, the rest of the household solid waste was piled into open spaces such as the banks of rivers or dumped directly into the river in Kisumu. We hypothesized that these uncollected municipal solid wastes would have directly or indirectly entered the water bodies. However, a certain amount of this portion would also have entered the soil; hence, the actual migration and transformation process and pathway were not further described in this study. Although limited to objective factors, these defects generally exist in the SFA studies [3] and it is difficult to avoid. Future studies should build more suitable models based on first-hand data, to more clearly and accurately characterize the food phosphorus flow in Kisumu and other cities in the Lake Victoria basin.

## 5. Conclusions

This study used substance flow analysis to quantitatively investigate the food phosphorus flow process in the Kisumu food system in Lake Victoria in 2023, and changes in the Kisumu food phosphorus footprint under different scenarios in 2030 were explored. In 2023, the main sources of phosphorus input into the Kisumu food system were fertilizer and food imports, while the phosphorus output was mainly animal and plant product exports. The runoff/leaching/erosion of the crop planting subsystem, waste loss of the household consumption subsystem, and blackwater loss of the pit latrine subsystem were the main pathways of phosphorus loss in the Kisumu food system, and these three subsystems were also the main contributors to the food phosphorus footprint of the city. Subsystems such as pit latrines and landfill were the key nodes for phosphorus accumulation in the Kisumu food system and have not been effectively utilized. Implementing strong governance measures in the upstream subsystem of the food system is the key to reducing the food phosphorus footprint in the city. The 2030 scenario analysis results indicate that the vegetarian scenario is the most effective single scenario solution for reducing the food phosphorus footprint in the future, while the implementation of combined scenarios, a comprehensive package of scenario solutions throughout the entire life cycle, from phosphorus source input to waste disposal, is the best choice for reducing phosphorus loss and suppressing an increase in food phosphorus footprint in the future. Our results indicate that Kisumu may enter a continuous upward trend with the improvement of socio-economic development of the city. Therefore, we propose a series of possible measures to reduce the food phosphorus footprint, including urine separation, installation of ST, proactive adjustment of dietary structure, especially reducing the consumption of animal-based products or at least maintaining phosphorus intake close to scientific recommendations, flexible layout of sanitary disposal facilities, and separation of organic and other waste streams in the city.

## Figures and Tables

**Table 1:** Classification of data uncertainties according to the data source and the level of specificity.

Level	Interval Factor	Information Source
1	×÷1.02	Kenya or Kisumu Bureau of Statistics; field survey in Kisumu; scientific literature/reports on Kenya/Kisumu
2	×÷1.03	Scientific literature/reports on African countries/tropical regions
3	×÷1.04	Scientific literature/reports on other parts of the world outside Africa/tropical regions
4	×÷1.07	Information provided by head of enterprise
5	×÷1.12	Estimates/values based on typical/average figures

Note: Adapted from [21,40].

**Table 2:** Scenario analysis information of food phosphorus footprint in Kisumu.

Scenario	Year	Subsystem	Focus	Population ^a^	Other Changes ^b^
0.Reference	2023	-	-	Current	None
1. BAU scenario	2030	All subsystem	population	Increase	None
2. Vegetarian scenario	2030	Household subsystem	Human behaviour	Increase	Vegetarian P uptake
3. Kitchen waste scenario	2030	Household subsystem	Collection	Increase	Improvement of kitchen waste collection rate
4. Urine separation scenario	2030	Pit latrine subsystem	Collection	Increase	Urine collected separately
5. Wastewater treatment scenario	2030	WWTP subsystem	Treatment	Increase	40% of P in sewage sludge recovered for agricultural use
6. Waste incineration scenario	2030	Landfill subsystem	Treatment	Increase	Recovery of P from solid waste incineration ash
7. Combined scenario	2030	All subsystem	Combined 1–6 scenario	Increase	Combined 1–6 scenario

Note: ^a^ The population data were obtained from KNBS [38]; ^b^ Assumptions based on the analysis in Section 2.5.

**Table 3:** Comparison of the food phosphorus footprints of Kisumu with other regions around the world.

Study	Local	Year	Scale	Method	Food Phosphorus Footprint (Kg P cap^−1^yr^−1^)
[16]	Brussels	2011	City	Based on SFA	7.70
[56]	Luxembourg	2007	Country	P-Calculator model	7.64
[56]	USA	2007	Country	P-Calculator model	6.09
[56]	Belgium	2007	Country	P-Calculator model	5.21
[11]	Japan	2013	Country	P-Calculator model	6.05
[11]	China	2013	Country	P-Calculator model	4.77
[11]	India	2013	Country	P-Calculator model	1.60
[15]	Indonesia	2013	Country	P-Calculator model	0.40
[14]	Rwanda	2020	Country	P-Calculator model	0.31
This study	Kisumu	2023	City	Based on SFA	0.67

## Data Availability

The original contributions presented in the study are included in the article; further inquiries can be directed to the corresponding author.

## Supplementary Materials

The following supporting information can be downloaded at: https://www.mdpi.com/article/10.3390/foods13142225/s1. Table S1. Parameters of phosphorus flow and stock calculations. Table S2. Equation of phosphorus flow model, output results and uncertainty. Refs. [66,67,68,69,70,71,72,73,74,75,76,77,78] are cited in Supplementary Material.

## References

[1] Cordell D., Drangert J.O., White S. (2009). The story of phosphorus: Global food security and food for thought. Glob. Environ. Chang..

[2] Cooper J., Carliell-Marquet C.A. (2013). Substance flow analysis of phosphorus in the UK food production and consumption system. Resour. Conserv. Recycl..

[3] Firmansyah I., Spiller M., Ruijter F.J.D., Carsjens G.J., Zeeman G. (2016). Assessment of nitrogen and phosphorus flows in agricultural and urban systems in a small island under limited data availability. Sci. Total Environ..

[4] Brownlie W.J., Sutton M.A., Reay D.S., Heal K.V., Spears B.M. (2021). Global actions for a sustainable phosphorus future. Nat. Food.

[5] UNEP (2024). Take Effective, Inclusive, and Sustainable Multilateral Actions to Address Climate Change, Biodiversity Loss, and Pollution.

[6] Kattel G., Reeves J., Western W.A., Zhang W.J., McGowan S., Cuo L., Scales P., Dowling K., He Q., Wang L. (2020). Healthy waterways and ecologically sustainable cities in Beijing-Tianjin-Hebei urban agglomeration (northern China): Challenges and future directions. WIREs Water.

[7] Bi J., Yuan Z., Zhang L., Chen Q. (2013). Quantifying Phosphorus Flow Pathways Through Socioeconomic Systems at the County Level in China. J. Ind. Ecol..

[8] Huang C., Gao B., Xu S., Huang Y., Yan X., Cui S. (2019). Changing phosphorus metabolism of a global aquaculture city. J. Clean. Prod..

[9] Jiang S., Hua H., Sheng H., Jarvie H.P., Liu X., Zhang Y., Yuan Z., Zhang L., Liu X. (2019). Phosphorus footprint in China over the 1961–2050 period: Historical perspective and future prospect. Sci. Total Environ..

[10] Hoekstra A.Y., Wiedmann T.O. (2014). Humanity’s unsustainable environmental footprint. Science.

[11] Oita A., Wirasenjaya F., Liu J., Webeck E., Matsubae K. (2020). Trends in the food nitrogen and phosphorus footprints for Asia’s giants: China, India, and Japan. Resour. Conserv. Recycl..

[12] Wang F., Sims J.T., Ma L., Ma W., Dou Z., Zhang F. (2011). The phosphorus footprint of China’s food chain: Implications for food security, natural resource management, and environmental quality. J. Environ. Qual..

[13] Elrys A.S., Desoky E.S.M., Ali A., Zhang J.B., Cheng Y. (2021). Sub-Saharan Africa’s food nitrogen and phosphorus footprints: A scenario analysis for 2050. Sci. Total Environ..

[14] Bizimana F., Dong W.X., Li X.X., Timilsina A., Zhang Y.M., Aluoch S.O., Qin S., Hu C.S. (2024). Estimating food nitrogen and phosphorus footprints and budgeting nitrogen and phosphorus flows of Rwanda’s agricultural food system during 1961–2020. Sci. Total Environ..

[15] Wirasenjaya F., Dhar A.R., Oita A., Matsubae K. (2023). Assessment of food-related nitrogen and phosphorus footprints in Indonesia. Sustain. Prod. Consum..

[16] Papangelou A., Towa E., Achten W.M.J., Mathijs E. (2021). A resource-based phosphorus footprint for urban diets. Environ. Res. Lett..

[17] Metson G.S., MacDonald G.K., Leach A.M., Compton J.E., Harrison J.A., Galloway J.N. (2020). The U.S. consumer phosphorus footprint: Where do nitrogen and phosphorus diverge?. Environ. Res. Lett..

[18] Grönman K., Ypyä J., Virtanen Y., Kurppa S., Soukka R., Seuri P., Finér A., Linnanen L. (2016). Nutrient footprint as a tool to evaluate the nutrient balance of a food chain. J. Clean. Prod..

[19] Joensuu K., Pulkkinen H., Kurppa S., Ypyä J., Virtanen Y. (2019). Applying the nutrient footprint method to the beef production and consumption chain. Int. J. Life Cycle Assess..

[20] Guo Z., Chen S.S., Kattel G.R., Mkumbo A.C., Xiong C., Gao Q., Shen Q. (2023). Scenario analysis of phosphorus flow in food production and consumption system in the Mwanza region, Tanzania. Sci. Total Environ..

[21] Laner D., Feketitsch J., Rechberger H., Fellner J. (2015). A Novel Approach to Characterize Data Uncertainty in Material Flow Analysis and its Application to Plastics Flows in Austria. J. Ind. Ecol..

[22] Alukwe I.A. (2015). Evaluating Implications of Vision 2030 on Nutrient Fluxes in Nairobi, Kenya. Am. J. Environ. Eng..

[23] Swilling M., Robinson B., Marvin S., Hodson M. (2013). City-Level Decoupling: Urban Resource Flows and the Governance of Infrastructure Transitions. Environ. Dev..

[24] UN-HABITAT (2016). Urbanization and Structural Transformation. Structural Transformation 2, Nairobi, Kenya. https://population.un.org/wup/.

[25] Watson V. (2009). Seeing from the South: Refocusing Urban Planning on the Globe’s Central Urban Issues. Urban Stud..

[26] UNEP/WAP (2012). UNEP/WAP: State of the Mediterranean Marine and Coastal Environment. United Nations Environment Programme/Mediterranean Action Plan. https://www.unep.org/resources/report/state-mediterranean-marine-and-coastal-environment-highlights-policy-makers.

[27] Regmi A., Dyck J. (2001). Effects of Urbanization on Global Food Demand. Changing Structure of Global Food Consumption and Trade.

[28] Mnthambala F., Tilley E., Tyrrel S., Sakrabani R. (2021). Phosphorus flow analysis for Malawi: Identifying potential sources of renewable phosphorus recovery. Resour. Conserv. Recycl..

[29] UN-WPP (2019). World Population Prospects: The 2019 Revision. United Nations, Department of Economic and Social Affairs, Population Division (2019). United Nations World Population Prospects. https://population.un.org/wpp/.

[30] Musungu P.C., Lalah J.O., Jondiko I.O., Ongeri D.M.K. (2014). The impact of nitrogenous and phosphorous nutrients from selected point sources in Kisumu City on River Kisat and Nyalenda Wigwa Stream before their discharge into Winam Gulf, Lake Victoria. Environ. Earth Sci..

[31] Kulekana A. (2008). Levels of nitrate and phosphate in some satellite lakes within the Lake Victoria basin, Tanzania. Tanzan. J. Sci..

[32] Zong Y., Chen S.S., Kattel G.R., Guo Z. (2023). Spatial distribution of non-point source pollution from total nitrogen and total phosphorous in the African city of Mwanza (Tanzania). Front. Environ. Sci..

[33] Matzinger A., Schmid M., Veljanoska-Sarafiloska E., Patceva S., Guseska D., Wagner B. (2007). Eutrophication of ancient Lake Ohrid: Global warming amplifies detrimental effects of increased nutrient inputs. Limnoloy Oceanogr..

[34] Odada E.O., Olago D.O., Kulindwa K., Ntiba M., Wandiga S.O. (2004). Mitigation of environmental problems in Lake Victoria, eastAfrica: Causal chain and policy options analyses. R. Swed. Acad. Sci..

[35] Cui S., Shi Y., Malik A., Lenzen M., Gao B., Huang W. (2016). A hybrid method for quantifying China’s nitrogen footprint during urbanisation from 1990 to 2009. Environ. Int..

[36] Stern D.I., Cleveland C.J. (2004). Environmental Kuznets Curve. Encyclopedia of Energy.

[37] CGK (County Government of Kisumu) (2022). Kisumu County Annual Development Plan 2021/2022.

[38] KNBS (Kenya National Bureau Statistical) (2023). Statistical Abstract 2023.

[39] Hedbrant J., Sörme L. (2001). Data Vagueness and Uncertainties in Urban Heavy-Metal Data Collection. Water Air Soil Pollut. Focus.

[40] Klinglmair M., Lemming C., Jensen L.S., Rechberger H., Astrupa T.F., Scheutz C. (2015). Phosphorus in Denmark: National and regional anthropogenic flows. Resour. Conserv. Recycl..

[41] Antikainen R., Lemola R., Nousiainen J.I., Sokka L., Esala M., Huhtanen P., Rekolainen S. (2005). Stocks and flows of nitrogen and phosphorus in the Finnish food production and consumption system. Agric. Ecosyst. Environ..

[42] City Facts. Population in Kisumu, Kenya 2030. https://www.city-facts.com/kisumu/population.

[43] Shinozaki N., Murakami K., Asakura K., Uechi K., Kobayashi S., Masayasu S., Sasaki S. (2018). Dietary phosphorus intake estimated by 4-day dietary records and two 24-hour urine collections and their associated factors in Japanese adults. Eur. J. Clin. Nutr..

[44] Oloko M., Capuano M.L., Ness B., Otiende F. Solid Waste Management: Recommendations for Kisumu, Kenya. Mistra Urban Futures Policy Brief, 2019:1. https://www.mistraurbanfutures.org/en/publication/solid-waste-management-recommendations-kisumu-kenya.

[45] Sibanda L.K., Obange N., Awuor F.O. (2017). Challenges of Solid Waste Management in Kisumu, Kenya. Urban Forum.

[46] Kalmykova Y., Harder R., Borgestedt H., Svanäng I. (2012). Pathways and management of phosphorus in urban areas. J. Ind. Ecol..

[47] Jonsson H., Stinzing A.R., Vinneras B. (2004). Guidelines on the Use of Urine and Faeces in Crop Production.

[48] CGK (County Government of Kisumu) (2019). Sanitation Polices Practices and Preferences in Kisumu, Kenya.

[49] Wu J.C., Franzén D., Malmström M.E. (2015). Anthropogenic phosphorus flows under different scenarios for the city of Stockholm, Sweden. Sci. Total Environ..

[50] West P.C., Gerber J.S., Engstrom P.M., Mueller N.D., Brauman K.A., Carlson K.M., Cassidy E.S., Johnston M., MacDonald G.K., Ray D.K. (2014). Leverage points for improving global food security and the environment. Science.

[51] Sumithra R., Thushyanthy M., Srivaratharasan T. (2013). Assessment of soil loss and nutrient depletion due to cassava harvesting: A case study from low input traditional agriculture. Int. Soil Water Conserv. Res..

[52] Cheng C.Y., Tsang C.K., Wong R.S.K., Chu L.M. (2011). Is Landfill Leachate a Potential Source of Nitrogen for Plant Growth?. International Conference on Environment and Industrial Innovation.

[53] Vila-Real C.P.M., Pimenta-Martins A.S., Kunyanga C.N., Mbugua S.K., Katina K., Maina N.H., Gomes A.M.P., Pinto E.C.B. (2022). Nutritional intake and food sources in an adulturban Kenyan population. Nutr. Bull..

[54] Boulanger P., Dudu H., Ferrari E., Mainar-Causapé A.J., Ramos M.P. (2020). Effectiveness of fertilizer policy reforms to enhance food security in Kenya: A macro–micro simulation analysis. Appl. Econ..

[55] Schönfeldt H.C., Hall N.G. (2012). Dietary protein quality and malnutrition in Africa. Br. J. Nutr..

[56] Metson G.S., Hale R.L., Iwaniec D.M., Cook E.M., Corman J.R., Galletti C.S., Childers D.L. (2012). Phosphorus in Phoenix: A budget and spatial representation of phosphorus in an urban ecosystem. Ecol. Appl. Publ. Ecol. Soc. Am..

[57] Santos A.F., Lopes D.V., Alvarenga P., Gando-Ferreira L.M., Quina M.J. (2024). Phosphorus removal from urban wastewater through adsorption using biogenic calcium carbonate. J. Environ. Manag..

[58] Garske B., Stubenrauch J., Ekardt F. (2024). Sustainable phosphorus management in European agricultural and environmental law. Rev. Eur. Comp. Int. Environ. Law.

[59] Bateman A., Horst D.V.D., Boardman D., Kansal A., Carliell-Marquet C. (2011). Closing the phosphorus loop in England: The spatio-temporal balance of phosphorus capture from manure versus crop demand for fertiliser. Resour. Conserv. Recycl..

[60] WHO (World Health Organization) (2013). Guidelines for the Safe Use of Wastewater, Excreta, and Grey Water.

[61] Mara D., Drangert J.O., Anh N.V., Tonderski A., Gulyas A., Tonderski K. (2007). Selection of sustainable sanitation arrangements. Water Policy.

[62] Kujawa-Roeleveld K., Fernandes T., Wiryawan Y., Tawfik A., Visser M., Zeeman G. (2005). Performance of UASB septic tank for treatment of concentrated black water within DESAR concept. Waterence Technol..

[63] Buathong T., Boontanon S.K., Boontanon N., Surinkul N., Harada H., Fujii S. (2013). Nitrogen Flow Analysis in Bangkok City, Thailand: Area Zoning and Questionnaire Investigation Approach. Procedia Environ. Sci..

[64] Roefs I., Meulman B., Vreeburg J.H.G., Spiller M. (2017). Centralised, decentralised or hybrid sanitation systems? Economic evaluation under urban development uncertainty and phased expansion. Water Res..

[65] Jensen P.D., Sullivan T., Carney C., Batstone D.J. (2014). Analysis of the potential to recover energy and nutrient resources from cattle slaughterhouses in Australia by employing anaerobic digestion. Appl. Energy.

[66] Erni M. (2015). Modelling Urban Water Flows: An Insight into Current and Future Water Availability and Pollution of a Fast-Growing City. Case Study of Kumasi, Ghana. (MSc.) Swiss Federal Institute of Technology, Zurich. http://e-collection.library.ethz.ch/eserv/eth:29522/eth-29522-01.pdf.

[67] FAO (2021). Cropland in Kisumu, Kenya (2021). Kenya, Nairobi. https://www.fao.org/3/cc0911en/cc0911en.pdf.

[68] FAO (2015). Tropical Livestock Unit (TLU). http://www.fao.org/family-farming/data-sources/dataportrait/livestock/en/.

[69] LVSWWDA (Lake Victoria South Water Works Development Agency) (2022). Kisumu County Water Master Plan Developed. Lake Victoria South Water Works Development Agency. https://www.lvswwda.go.ke/kisumu-county-water-master-plan-developed/.

[70] Mbwele L. (2006). Microbial Phosphorus Removal in Waste Stabilization Pond Wastewater Treatment Systems. Ph.D. Thesis.

[71] NRC (2000). Nutrient Requirements of Beef Cattle, Update 2000.

[72] NRC (2007). Nutrient Requirements of Small Ruminants: Sheep, Goats, Cervids, and New World Camelids. National Research Council. Committee on Nutrient Requirements of Small Ruminants. https://nap.nationalacademies.org/catalog/11654/nutrient-requirements-of-small-ruminants-sheep-goats-cervids-and-new.

[73] NRP (1994). National Research Council. Nutrient Requirements of Poultry: Ninth Revised Edition.

[74] Stadlmayr B., Charrondiere U.R., Enujiugha V.N., Bayili R.G., Fagbohoun E.G., Samb B. (2012). West African Food Composition Table-Table de Composition des Aliments d’Afrique de l’Ouest. FAO, Rome. https://www.academia.edu/20337868/Nutrient_Requirements_of_Beef_Cattle_Kebutuhan_Nutrisi_Sapi.

[75] Wang X.Y. (2017). The Flow of Nitrogen, Phosphorus, and Carbon in the Food Production and Consumption System of Kunming City and Its Environmental Load. Master’s Thesis.

[76] WEDC (2016). SFD Promotion Initiative Kisumu Kenya. 2016. Kenya, Nairobi. https://www.susana.org/_resources/documents/default/3-2622-7-1471944664.pdf.

[77] Zheng W.T. (2018). Research on Resource Utilization of Crop Straw in Henan Province. Ph.D. Thesis.

[78] ONEP (2010). Depollution de la 2010 Marchica. Assainissement du Grand Nador, Interception, Transfert et Epuration des Eaux Usées. Extension des reseaux d’assainissement liquide des Municipalités et Centres du Grand Nador. Royaume du Maroc. Office National de l’Eau Potable. http://www.onep.ma/news/2010/In_Nador_31-05-2010/plaquette_Ass.GdNADOR_28-05-2010.pdf.

