# Supplementary material for: Scenario Analysis of Food Phosphorus Footprint in Kisumu, a Lakeside East African City in Lake Victoria (Kenya)
Source: Foods. 2024 Jul 16;13(14):2225. doi: 10.3390/foods13142225 (PMC11276245; doi:10.3390/foods13142225)
Supplement: Supplementary file 1 [file foods-13-02225-s001.zip › foods-3091921-supplementary.pdf]

Scenario Analysis of Food Phosphorus Footprint in Kisumu, A Lakeside East African City in Lake Victoria (Kenya)

Zheng Guo [1], Sophia Shuang Chen [1,2,*], Giri Raj Kattel [1,3,4], Wenyi Qiao [1], Linglong Lu [1], Rong Li [1] and Anna Charles Mkumbo [5]

1 School of Geographical Sciences, Nanjing University of Information Science &Technology, Nanjing 210044, China; 003583@nuist.edu.cn (Z.G.); gkattel@unimelb.edu.au (G.R.K.); qiaowenyidl@163.com (W.Q.); 202183670017@nuist.edu.cn (L.L.); lirong020129@163.com (R.L.)

2 Key Laboratory of Watershed Geographic Sciences, Nanjing Institute of Geography and Limnology, Chi-nese Academy of Sciences, Nanjing 210008, China

3 Department of Infrastructure Engineering, The University of Melbourne, Melbourne 3052, Australia

4 Department of Hydraulic Engineering, Tsinghua University, Beijing 100190, China

5 Tanzania Fisheries Research Institute, Dar es Salaam P.O. Box 750, Tanzania; annamkumbo49@gmail.com

* Correspondence: schens@niglas.ac.cn

Contents:

Table S1 Parameters of phosphorus flow and stock calculations

Table S2 Equation of phosphorus flow model, output results and uncertainty

Table S1 Parameters of phosphorus flow and stock calculations

Parameters	Description	Value	Unit	Data Source	Year	Level
$n_{population}$	Number of totals inhabits of Kisumu	1248474	inhabitant	[38]	2023	1
n_{animal_cattle}	Number of cattle	297757	head	[38]	2015	1
n_{animal_goat}	Number of goat	244155	head	[38]	2015	1
n_{animal_sheep}	Number of sheep	240147	head	[38]	2015	1
n_{animal_pig}	Number of pig	5124	head	[38]	2015	1
$n_{animal\_poultry}$	Number of poultry	1312828	head	[38]	2015	1
$n_{feeding\ days\_cattle}$	Average feeding days of cattle	365	days	Interview with farmers, 2023	2023	1
$n_{feeding\ days\_goat}$	Average feeding days of goat	365	days	Interview with farmers, 2023	2023	1
$n_{feeding\ days\_sheep}$	Average feeding days of sheep	365	days	Interview with farmers, 2023	2023	1
$n_{feeding\ days\_pig}$	Average feeding days of pig	240	days	Interview with farmers, 2023	2023	1
$n_{feeding\ days\_poultry}$	Average feeding days of poultry	284	days	Interview with farmers, 2023	2023	1
n_{milk}	Milk production	26255800	kg	[38]	2023	1
$n_{poultry\ egg}$	Poultry egg production	1105104	kg	[38]	2023	1
n_{crop_cereal}	Cereal yield	855083000	kg	[38]	2015	1
n_{crop_root}	Root yield	29595000	kg	[38]	2015	1
n_{crop_legume}	Legume yield	100629000	kg	[38]	2015	1

$n_{crop\_vegetable}$	Vegetable yield	12897000	kg	[38]	2015	1
n_{crop_fruit}	Fruit yield	1633289000	kg	[38]	2015	1
$n_{crop\ food\_cereal}$	Amount of cereal food consumed	59.04	kg (cap year)$^{-1}$	Questionnaire survey of residents, 2023	2023	1
$n_{crop\ food\_root}$	Amount of root food consumed	14.88	kg (cap year)$^{-1}$	Questionnaire survey of residents, 2023	2023	1
$n_{crop\ food\_legume}$	Amount of legume food consumed	14.87	kg (cap year)$^{-1}$	Questionnaire survey of residents, 2023	2023	1
$n_{crop\ food\_vegetable}$	Amount of vegetable food consumed	25.2	kg (cap year)$^{-1}$	Questionnaire survey of residents, 2023	2023	1
$n_{crop\ food\_fruit}$	Amount of fruit food consumed	33.24	kg (cap year)$^{-1}$	Questionnaire survey of residents, 2023	2023	1
$n_{animal\ food\_beef}$	Amount of beef food consumed	13.92	kg (cap year)$^{-1}$	Questionnaire survey of residents, 2023	2023	1
$n_{animal\ food\_goat\ meat}$	Amount of goat meat food consumed	4.14	kg (cap year)$^{-1}$	Questionnaire survey of residents, 2023	2023	1
$n_{animal\ food\_pork}$	Amount of pork food consumed	4.08	kg (cap year)$^{-1}$	Questionnaire survey of residents, 2023	2023	1

$n_{animal\ food\_poulyry\ meat}$	Amount of poultry meat food consumed	12.06	kg (cap year)$^{-1}$	Questionnaire survey of residents, 2023	2023	1
$n_{animal\ food\_egg}$	Amount of poultry egg food consumed	15.72	kg (cap year)$^{-1}$	Questionnaire survey of residents, 2023	2023	1
$n_{animal\ food\_milk}$	Amount of milk food consumed	37.5	kg (cap year)$^{-1}$	Questionnaire survey of residents, 2023	2023	1
$n_{P,human\ manure}$	Amount of P produced by human faeces	0.1	kg P (cap year)$^{-1}$	[47]	2004	2
$n_{P,human\ urine}$	Amount of P produced by human urine	0.3	kgP (cap year)-1	[47]	2004	2
$n_{manure\ animal\_cattle}$	Production of cattle manure	18	kg/day	[75]	2017	3
$n_{manure\ animal\_goat}$	Production of goat manure	1.5	kg/day	[75]	2017	3
$n_{manure\ animal\_sheep}$	Production of sheep manure	1.5	kg/day	[75]	2017	3
$n_{manure\ animal\_pit}$	Production of pit manure	2	kg/day	[75]	2017	3
$n_{manure\ animal\_poultry}$	Production of poultry manure	0.13	kg/day	[75]	2017	3
$n_{urine\ animal\_cattle}$	Production of cattle urine	9	kg/day	[75]	2017	3
$n_{urine\ animal\_goat}$	Production of goat urine	0.5	kg/day	[75]	2017	3
$n_{urine\ animal\_sheep}$	Production of sheep urine	0.5	kg/day	[75]	2017	3
$n_{urine\ animal\_pit}$	Production of pit urine	3	kg/day	[75]	2017	3
$n_{urine\ animal\_poultry}$	Production of poultry urine	0	kg/day	[75]	2017	3

$n_{kitchen\ waste}$	The amount of kitchen waste generated	120	kg/year	[75]	2017	3
$n_{P,human\ urine}$	amount of P produced by human feces	0.1	kg P (cap year)$^{-1}$	[47]	2004	2
$n_{P,human\ urine}$	amount of P produced by human urine	0.3	kg P (cap year)$^{-1}$	[47]	2004	2
$n_{domestic\ sewage}$	Urban domestic sewage production	6488888889	L/year	[69]	2022	4
$n_{domestic\ sewage\_WWTPS}$	Urban domestic sewage treatment capacity	2920000000	L/year	[69]	2022	4
$n_{landfill\ leachate}$	The amount of leachate generated from landfill sites	0.027	L (kg waste)$^{-1}$ year^{-1}	[41]	2005	3
$a_{P,fertiliazer\_arable}$	Amount of P in fertilizer application in arable land	15.95	kg ha^{-1} year^{-1}	[54]	2014	1
$a_{P,requirement\_cattle}$	P requirement for cattle growth	13	gP head^{-1} day^{-1}	[71]	2000	3
$a_{P,requirement\_goat}$	P requirement for goat growth	1	gP head^{-1} day^{-1}	[72]	2007	3
$a_{P,requirement\_sheep}$	P requirement for sheep growth	1.3	gP head^{-1} day^{-1}	[72]	2007	3
$a_{P,requirement\_pig}$	P requirement for pig growth	3.4	gP head^{-1} day^{-1}	[72]	2007	3
$a_{P,requirement\_poultry}$	P requirement for poultry growth	0.89	gP head^{-1} day^{-1}	[73]	1994	3
$C_{f,feed\_cattle}$	Correction factors of nutritional quality for cattle growth	0.6	-	[68]	2015	2

$C_{f, feed\_goat}$	Correction factor of nutritional quality required for goat growth	1	-	[68]	2015	2
$C_{f, feed\_sheep}$	Correction factor of nutritional quality required for sheep growth	0.8	-	[68]	2015	2
$C_{f, feed\_pig}$	Correction factor of nutritional quality required for pig growth	0.8	-	[68]	2015	2
$C_{f, feed\_poultry}$	Correction factor of nutritional quality required for poultry growth	0.2	-	[68]	2015	2
$C_{P, body\_cattle}$	P content of cattle body	1.74	gP/kg	[74]	2012	2
$C_{P, body\_goat}$	P content of goat body	1.50	gP/kg	[74]	2012	2
$C_{P, body\_sheep}$	P content of sheep body	1.50	gP/kg	[74]	2012	2
$C_{P, body\_pig}$	P content of pig body	1.89	gP/kg	[74]	2012	2
$C_{P, body\_poultry}$	P content of poultry body	1.46	gP/kg	[74]	2012	2
$C_{P, manure\_cattle}$	The proportion of phosphorus in cattle manure	0.218	%	[75]	2017	3
$C_{P, manure\_goat}$	The proportion of phosphorus in goat manure	0.495	%	[75]	2017	3
$C_{P, manure\_sheep}$	The proportion of phosphorus in sheep manure	0.495	%	[75]	2017	3

$C_{P,manure\_pig}$	The proportion of phosphorus in pig manure	0.563	%	[75]	2017	3
$C_{P, manure\_poultry}$	The proportion of phosphorus in poultry manure	0.757	%	[75]	2017	3
$C_{P, urine\_cattle}$	The proportion of phosphorus in cattle urine	0.039	%	[75]	2017	3
$C_{P, urine\_goat}$	The proportion of phosphorus in goat urine	0.048	%	[75]	2017	3
$C_{P, urine\_sheep}$	The proportion of phosphorus in sheep urine	0.048	%	[75]	2017	3
$C_{P, urine\_pig}$	The proportion of phosphorus in pig urine	0.05	%	[75]	2017	3
$C_{P, urine\_poultry}$	The proportion of phosphorus in poultry urine	0	%	[75]	2017	3
$C_{P, milk}$	P content of milk	1.06	gP (kg product)$^{-1}$	[74]	2012	2
$C_{P, meat\_cattle}$	P content of poultry egg	1.98	gP (kg product)$^{-1}$	[74]	2012	2
$C_{P, meat\_cattle}$	P content of cattle meat	1.74	gP (kg product)$^{-1}$	[74]	2012	2

$C_{P,\ meat\_goat}$	P content of goat meat	1.50	gP (kg product)$^{-1}$	[74]	2012	2
$C_{P,\ meat\_sheep}$	P content of sheep meat	1.50	gP (kg product)$^{-1}$	[74]	2012	2
$C_{P,\ meat\_pig}$	P content of pig meat	1.89	gP (kg product)$^{-1}$	[74]	2012	2
$C_{P,\ meat\_poultry}$	P content of poultry meat	1.46	gP (kg product)$^{-1}$	[74]	2012	2
$C_{P,\ crop\_cereal}$	P content of cereal crop	2.22	gP (kg product)$^{-1}$	[74]	2018	2
$C_{P,\ crop\_root}$	P content of root crop	0.49	gP (kg product)$^{-1}$	[74]	2012	2
$C_{P,\ crop\_legume}$	P content of legume crop	0.56	gP (kg product)$^{-1}$	[74]	2012	2
$C_{P,\ crop\_vegetable}$	P content of vegetable crop	0.39	gP (kg product)$^{-1}$	[74]	2012	2
$C_{P,\ crop\_fruit}$	P content of fruit crop	0.18	gP (kg product)$^{-1}$	[74]	2012	2
$C_{P,\ straw\_legume}$	P content of legume straw	2	gP (kg product)$^{-}$	[74]	2010	2

			1			
$C_{P, food\_cereal}$	P content of cereal food	2.22	gP (kg product)$^{-1}$	[74]	2012	2
$C_{P, food\_root}$	P content of root food	0.49	gP (kg product)$^{-1}$	[74]	2012	2
$C_{P, food\_legume}$	P content of legume food	0.56	gP (kg product)$^{-1}$	[74]	2012	2
$C_{P, food\_vegetable}$	P content of vegetable food	0.39	gP (kg product)$^{-1}$	[74]	1995	2
$C_{P, food\_fruit}$	P content of fruit food	0.18	gP (kg product)$^{-1}$	[74]	1995	2
$C_{P, food\_beef}$	P content of beef food	1.74	gP (kg product)$^{-1}$	[74]	2012	2
$C_{P, food\_goat\ meat}$	P content of goat meat food	1.50	gP (kg product)$^{-1}$	[74]	2012	2
$C_{P, food\_mutton}$	P content of mutton food	1.50	gP (kg product)$^{-1}$	[74]	2012	2
$C_{P, food\_pork}$	P content of pork food	1.89	gP (kg product)$^{-1}$	[74]	2012	2

$C_{P, food\_poultry\ meat}$	P content of poultry food	1.46	gP (kg product)$^{-1}$	[74]	2012	2
$C_{P, food\_poultry\ egg}$	P content of poultry egg food	1.98	gP (kg product)$^{-1}$	[74]	2012	2
$C_{P, food\_milk}$	P content of milk food	1.06	gP (kg product)$^{-1}$	[74]	2012	2
$C_{P, dry\ material}$	P content of dry material in kitchen waste	5.2	gP (kg product)$^{-1}$	[3]	2016	2
$C_{P,domestic\ sewage\ in}$	Phosphorus content in wastewater collected by sewage treatment plants before treatment	7	mg/L	[78]	2019	2
$C_{P,domestic\ sewage\ out}$	Phosphorus content in wastewater collected by sewage treatment plants after treatment	3.85	mg/L	[70]	2006	1
$C_{P,leachate}$	Phosphorus content in leachate from landfill	4.5	mg/L	[66]	2007	2
$Frac_{P,\ leaching/runoff/erosion}$	fraction of P loss by leaching/erosion/runoff processes	1.99	kgP ha^{-1}	[51]	2013	2
$r_{sell\ \_\ cattle}$	Ratio of cattle sell	59.85	%	Interview with farmers, 2019	2019	1

r_{sell_goat}	Ratio of goat sell	51.23	%	Interview with farmers, 2019	2019	1
r_{sell_sheep}	Ratio of sheep sell	51.23	%	Interview with farmers, 2019	2019	1
r_{sell_pig}	Ratio of pig sell	68.33	%	Interview with farmers, 2019	2019	1
$r_{sell\_poultry}$	Ratio of poultry sell	85.25	%	Interview with farmers, 2019	2019	1
$r_{S\&P\_legume}$	Ratio of straw and product in legume crop	1	-	[77]	2018	3
$r_{legum\ straw\ cut}$	Ratio of legume crop straw cut	2/3	-	Interview with farmers, 2023	2023	1
$r_{dry\ material}$	Ratio of dry material in kitchen waste	40	%	[3]	2016	2
$r_{kitchen\ waste\_collection}$	Ration of kitchen waste collection	20	%	[45]	2017	1
$r_{kitchen\ waste\_loss}$	Ratio of kitchen waste loss	80	%	[45]	2017	1
$r_{domestic\ sewage\_loss}$	Ration of domestic sewage loss	55	%	Expert estimate	2023	5
$r_{blackwater\_loss}$	Ration of blackwater loss	34	%	Expert estimate	2023	5
$r_{blackwater\_pit\ latrines}$	Ratio of blackwater entering pit latrines	66	%	[76]	2016	1
$r_{blackwater\_overflow\&discharge}$	Ration of blackwater entering the water body by overflow & discharge from pit latrines	50	%	[76]	2015	1
$r_{human\ manure\_farmland}$	Ratio of human manure return to farmland	33	%	[76]		1
$r_{animal\ manure\_farmland}$	Ratio of animal manure returning farmland	60	%	Expert estimate, 2019	2019	5

$r_{animal\ manure\_loss}$	Ratio of animal manure loss	30	%	Expert estimate, 2019	2019	5
$r_{animal\ manure\_stock}$	Ratio of animal manure stock	10	%	Expert estimate, 2019	2019	5
$r_{P,landfill}$	Ratio of P loss in landfill	20	%	[52]	2011	3
S_{area}	Total area of cultivated land in Kisumu	106447.4	ha	[67]	2021	1
W_{body_cattle}	Average live weight of cattle before slaughter	250	kg head^{-1}	[68]	2015	2
W_{body_goat}	Average live weight of goat before slaughter	30	kg head^{-1}	[68]	2015	2
W_{body_sheep}	Average live weight of sheep before slaughter	30	kg head^{-1}	[68]	2015	2
W_{body_pig}	Average live weight of pig before slaughter	168	kg head^{-1}	[68]	2015	2
$W_{body\_poultry}$	Average live weight of poultry before slaughter	2.09	kg head^{-1}	[73]	1994	3

Table S2 Equation of P flow model, output results and uncertainty

Crop planting subsystem	Average	Upper and lower limits of flow	Reconciled value in STAN

				and stock		
Flow	(tP/year)	(tP/year)	t P yr-1	t P yr-1	t P yr-1	uncertainty (%)
F1	Fertilizer application	$F1=S_{area}\times a_{P,fertilizer\_arable}$	1697.84	[1638.75,1759.35]	1697.84	3.6
F2	Crop product output	$F2=\sum_{i=1}^{5} n_{crop\_i}\times C_{P,crop\_i}-\sum_{i=1}^{5} n_{crop\ food\_i}\times C_{P,crop\ food\_i}\times n_{population}$	2065.28	[2005.13,2127.24]	2065.28	3
F3	Runoff/leaching/erosion	$S_{area}\times Frac_{P,Leaching/runoff/erosion}$	211.83	[204.46,219.47]	211.83	3.6
F4	Straw feed	$F4=n_{legum}\times r_{S\&P\_legum}\times r_{legum\ straw\ cut}\times C_{P,legum}$	134.17	[119.06,151.20]	134.17	12.7
F5	Crop product	$F5=\sum_{i=1}^{5} n_{crop\ food\_i}\times C_{P,crop\ food\_i}\times n_{population}$	202.88	[196.97,208.96]	202.88	3
S1	Stock1	$S1=F1+F6+F12-F2-F3-F4-F5$	-803.73	[-825.59,-779.62]		
Animal production subsystem			Average	Upper and lower limits of flow and stock	Reconciled value in STAN	
Flow	Description	Equation	t P yr-1	t P yr-1	t P yr-1	uncertainty (%)
F6	Animal manure	$F6=\sum_{i=1}^{5} n_{animal\_i}\times\sum_{i=1}^{5}(n_{manure\ animal\_i}+n_{urine\ animal\_i})\times n_{feeding\ days\_i}\times r_{animal\text{-}manure\_farm}$	3.83	[3.45,4.25]	3.83	11

F7	Animal feed	$F7=(\sum_{i=1}^{5} n_{animal\_i} \times (1-r_{sell\_i}) \times n_{feeding\ days\_i} \times a_{P,requirement\_i} \times C_{f,feed\_i} + \sum_{i=1}^{5} n_{an}$[illegible] $\times r_{sell\_i} \times W_{body\_i} \times C_{P,body\_i} + n_{milk} \times C_{P,milk} + n_{poultry\ egg} \times C_{P,poultry\ egg}) - F4$	428.19	[415.72,441.04]	428.19	3
F8	Animal product output	$F8 = (F4 + F7 - F6 - F9 - F10 - S2)$	397.89	[365.04,433.70]	397.89	9
F9	Animal products	$F9=\sum_{i=1}^{5} n_{animal\ food\_i} \times C_{P,animal\ food\_i} \times n_{population}$	158.09	[154.99,161.25]	158.09	2
F10	Animal manure loss	$F10 = \sum_{i=1}^{5} n_{animal\_i} \times \sum_{i=1}^{5} (n_{manure\ animal\_i} + n_{urine\ animal\_i}) \times n_{feeding\ days\_i} \times r_{animal\ manure\_loss}$	1.92	[1.73,2.13]	1.92	11
S2	Stock2	$S2 = \sum_{i=1}^{5} n_{animal\_i} \times \sum_{i=1}^{5} (n_{manure\ animal\_i} + n_{urine\ animal\_i}) \times n_{feeding\ days\_i} \times r_{animal\ manure\_stock}$	0.64	[0.58,0.71]	0.64	11
Household consumption subsystem			Average	Upper and lower limits of flow and stock	Reconciled value in STAN	
Flow	Description	Equation	t P yr^{-1}	t P yr^{-1}	t P yr^{-1}	uncertainty (%)
F11	Food products	$F11=F12+F13+F14+F15+F17-F9-F5$	604.24	[539.50,676.74]	604.24	8.6
F12	Human manure	$F12 = n_{population} \times (n_{P,human\ fmanure} + n_{P,human\ urine}) \times r_{human\ manure\_farmland}$	108.77	[98.88,119.64]	108.77	9.9
F13	Kitchen waste	$F13=n_{population} \times n_{kitchen\ waste} \times r_{dry\ material} \times C_{P,kitchen\ waste} \times r_{kitchen\ waste\ collection}$	62.32	[57.36,67.72]	62.32	8.6

F17	Domestic sewage	$F17 = n_{domestic\ sewage\_WWTPS} \times C_{P,domestic\ sewage\ in}$	20.44	[19.55,21.35]	20.44	4.5
F14	Domestic waste loss	$F14{=}F13 \times r_{kitchen\ waste\_loss} + F15 \times r_{blackwater\_loss} + n_{domestic\ sewage} \times r_{domestic\ sewage\_loss} \times C_{P,WWin}$	444.07	[411.18,479.60]	444.07	7.3
Landfill subsystem			Average	Upper and lower limits of flow and stock	Reconciled value in STAN	
Flow	Description	Equation	t P yr^{-1}	t P yr^{-1}	t P yr^{-1}	uncertainty (%)
F19	Leachate	$F19{=}n_{population} \times n_{kitchen\ waste} \times n_{landfill\ \ leachate} \times C_{P,leachate}$	0.02	[0.017,0.019]	0.02	5.6
S4	Stock4	$S4 = F13 - F19$	62.32	[57.16,67.91]	62.32	8.6
Pit latrines subsystem			Average	Upper and lower limits of flow and stock	Reconciled value in STAN	
Flow	Description	Equation	t P yr^{-1}	t P yr^{-1}	t P yr^{-1}	uncertainty (%)
F15	Blackwater	$F15{=}n_{population} \times (n_{P,human\ fmanure} + n_{P,human\ urine}) \times r_{balckwater\_pit\ latrines}$	329.60	[299.63,362.56]	329.60	7.8

F16	Blackwater discharge	$F16{=}F15\times r_{blackwater\_overflow\&discharge}$	164.80	[149.55,181.61]	164.80	10.2
S5	Stock5	$S5{=}F15{-}F16$	164.80	[140.85.192.81]	164.80	10.2
WWTPs subsystem			Average	Upper and lower limits of flow and stock	Reconciled value in STAN	
Flow	Description	Equation	t P yr^{-1}	t P yr^{-1}	t P yr^{-1}	uncertainty (%)
F18	Treated sewage discharge	$F18{=}n_{domestic\ sewage\_WWTPS}\times C_{P,domestic\ sewage\ out}$	11.24	[10.85,11.65]	11.24	3.6
S6	Stock6	$Stock6{=}F20-F21$	9.20	[8.60,9.84]	9.20	7

Reference

3. Firmansyah, I.; Spiller, M.; Ruijter, F.J.D.; Carsjens, G.J.; Zeeman, G. Assessment of nitrogen and phosphorus flows in agricultural and urban systems in a small island under limited data availability. Sci. Total Environ. **2016,** 574, 1521–1532. https://doi.org/10.1016/j.scitotenv.2016.08.159..

38.KNBS (Kenya National Bureau Statistical). Statistical Abstract 2023; KNBS: Nairobi, Kenya, **2023.** Available online: https://www.knbs.or.ke/wp-content/uploads/2023/12/2023-Statistical-Abstract-Final-PDF_2.pdf (accessed on 5 October 2023).

41.Antikainen, R.; Lemola, R.; Nousiainen, J.I.; Sokka, L.; Esala, M.; Huhtanen, P.; Rekolainen, S. Stocks and flows of nitrogen and phosphorus in the Finnish food production and consumption system. Agric. Ecosyst. Environ. **2005,** 107, 287–305. https://doi.org/10.1016/j.agee.2004.10.025.

45.Sibanda, L. K.; Obange, N.; Awuor, F. O. Challenges of Solid Waste Management in Kisumu, Kenya. Urban Forum, **2017,** 28, 387–402.https://doi.org/10.1007/s12132-017-9316-1

47. Jonsson, H.; Stinzing, A.R.; Vinneras, B. Guidelines on the Use of Urine and Faeces in Crop Production; EcoSanRes Publication Series; Stockholm Environment Institute: Stockholm, Sweden, **2004.**

Available online: http://www.ecosanres.org/pdf_files/ESR-factsheet-06.pdf (accessed on 2 October 2023).

51.Sumithra, R.; Thushyanthy, M.; Srivaratharasan, T. Assessment of soil loss and nutrient depletion due to cassava harvesting: A case study from low input traditional agriculture. Inter. Soil and Water Conser. Res, **2013,** 1, 72–79.https://doi.org/10.1016/S2095-6339(15)30041-1

52. Cheng, C.Y.; Tsang, C.K.; Wong, R.S.K.; Chu, L.M. Is Landfill Leachate a Potential Source of Nitrogen for Plant Growth? In *International Conference on Environment and Industrial Innovation*; IACSIT Press, Singapore, **2011**; Volume 12, pp. 286–295. Available online: https://www.researchgate.net/publication/268292676_Is_Landfill_Leachate_a_Potential_Source_of_Nitrogen_for_Plant_Growth (accessed on 4 October 2023).

54.Boulanger, P.; Dudu, H.; Ferrari, E.; Mainar-Causapé, A.J.; Ramos, M.P. Effectiveness of fertilizer policy reforms to enhance food security in Kenya: A macro–micro simulation analysis. Appl. Econ. **2020,** 54, 841–861. https://doi.org/10.1080/00036846.2020.1808180.

66.Erni, M, . Modelling urban water flows: An insight into current and future water availability and pollution of a fast-growing city. Case study of Kumasi, Ghana. (MSc.) Swiss Federal Institute of Technology, Zurich, **2015.** Available online: http://e-collection.library.ethz.ch/eserv/eth:29522/eth-29522-01.pdf (accessed on 2 August 2023).

67.FAO. Cropland in Kisumu, Kenya (2021). Kenya, Nairobi, **2021.**Available online: https://www.fao.org/3/cc0911en/cc0911en.pdf.(accessed on 2 October 2023).

68.FAO. Tropical Livestock Unit (TLU), **2015.** Available online: http://www.fao.org/family-farming/data-sources/dataportrait/livestock/en/ (accessed on 2 October 2023).

69.LVSWWDA (Lake Victoria South Water Works Development Agency). Kisumu county water master plan developed. Lake Victoria South Water Works Development Agency, **2022.** Available online: https://www.lvswwda.go.ke/kisumu-county-water-master-plan-developed/(accessed on 2 October 2023).

70.Mbwele, L. Microbial Phosphorus Removal in Waste Stabilization Pond Wastewater Treatment Systems. Phd Thesis: School of Biotechnology, Royal Institute of Technology, Stockholm, Sweden, **2006.** Available online : https://www.semanticscholar.org/paper/Microbial-Phosphorus-Removal-in-Waste-Stabilisation-Mbwele/11a694e77c927cb1f65889945ccb77de5b4f02a4(accessed on 5 October 2023).

71.NRC. Nutrient Requirements of Beef Cattle, Update **2000.** National Academy Press, Washington, DC: The National Academies Press, 2000. Available online: https://nap.nationalacademies.org/catalog/9791/nutrient-requirements-of-beef-cattle-seventh-revised-edition-update-2000(accessed on 5 October 2023).

72.NRC. Nutrient requirements of small ruminants: sheep, goats, cervids, and new world camelids. National Research Council. Committee on Nutrient Requirements of Small Ruminants, **2007.**Available online: https://nap.nationalacademies.org/catalog/11654/nutrient-requirements-of-small-ruminants-sheep-goats-cervids-and-new (accessed on 15 October 2023).

73.NRP. National Research Council. Nutrient Requirements of Poultry: Ninth Revised Edition. Washington, DC: The National Academies Press, **1994.** Available online: https://nap.nationalacademies.org/catalog/2114/nutrient-requirements-of-poultry-ninth-revised-edition-1994 (accessed on 15 October 2023).

74.Stadlmayr, B.; Charrondiere, U. R.; Enujiugha, V. N.; Bayili, R. G.; Fagbohoun, E. G.; Samb, B. West African Food Composition Table-Table de composition des aliments d'Afrique de l'Ouest. FAO, Rome, **2012.** Available online: https://www.academia.edu/20337868/Nutrient_Requirements_of_Beef_Cattle_Kebutuhan_Nutrisi_Sapi(accessed on 12 October 2023).

75.Wang, X. Y. The Flow of Nitrogen, Phosphorus, and Carbon in the Food Production and Consumption System of Kunming City and Its Environmental Load. Master Thesis:Nanjing, Nanjing Normal University, China, 2017.Available online: https://xueshu.baidu.com/usercenter/paper/show?paperid=1r6202508m2u06h0cf350rf0h7028927(accessed on 12 October 2023).

76.WEDC. SFD Promotion Initiative Kisumu Kenya. 2016. Kenya, Nairobi, **2016.** Available online: https://www.susana.org/_resources/documents/default/3-2622-7-1471944664.pdf (accessed on 20 October 2023).

77.Zheng, W. T. Research on resource utilization of crop straw in Henan province. PhD Thesis: Henan Normal University, Zhengzhou, China, **2018.** (in Chinese). Available online: https://xueshu.baidu.com/usercenter/paper/show?paperid=1k3c0xm0bp170pc0bb7x0ca0dt770858&site=xueshu_se (accessed on 20 October 2023).
80.ONEP. Depollution de la 2010 Marchica. Assainissement du Grand Nador, Interception, Transfert et Epuration des Eaux Usées. Extension des reseaux d'assainissement liquide des Municipalités et Centres du Grand Nador. Royaume du Maroc. Office National de l'Eau Potable, **2010.** Available online: http://www.onep.ma/news/2010/In_Nador_31-05-2010/plaquette_Ass.GdNADOR_28-05-2010.pdf(accessed on 15 October 2023).
